# Insights into the Response of Perennial Ryegrass to Abiotic Stress: Underlying Survival Strategies and Adaptation Mechanisms

**DOI:** 10.3390/life12060860

**Published:** 2022-06-08

**Authors:** Cuicui Miao, Yuting Zhang, Xuechun Bai, Tao Qin

**Affiliations:** College of Grassland Agriculture, Northwest A&F University, Yangling District, Xianyang 712100, China; a18645294327@nwafu.edu.cn (C.M.); yuting.zhang@nwafu.edu.cn (Y.Z.); bxc1121195605@nwsuaf.edu.cn (X.B.)

**Keywords:** *Lolium perenne* L., drought, salt, extreme temperature, stress resistance gene, molecular mechanism

## Abstract

Perennial ryegrass (*Lolium perenne* L.) is an important turfgrass and gramineous forage widely grown in temperate regions around the world. However, its perennial nature leads to the inevitable exposure of perennial ryegrass to various environmental stresses on a seasonal basis and from year to year. Like other plants, perennial ryegrass has evolved sophisticated mechanisms to make appropriate adjustments in growth and development in order to adapt to the stress environment at both the physiological and molecular levels. A thorough understanding of the mechanisms of perennial ryegrass response to abiotic stresses is crucial for obtaining superior stress-tolerant varieties through molecular breeding. Over the past decades, studies of perennial ryegrass at the molecular and genetic levels have revealed a lot of useful information to understand the mechanisms of perennial ryegrass adaptation to an adverse environment. Unfortunately, molecular mechanisms by which perennial ryegrass adapts to abiotic stresses have not been reviewed thus far. In this review, we summarize the recent works on the genetic and molecular mechanisms of perennial ryegrass response to the major abiotic stresses (i.e., drought, salinity, and extreme temperatures) and discuss new directions for future studies. Such knowledge will provide valuable information for molecular breeding in perennial ryegrass to improve stress resistance and promote the sustainability of agriculture and the environment.

## 1. Introduction

Drought, high salinity, and extreme temperatures are major adverse environmental stresses that plants often encounter, which is further complicated by the potential impact of climate change. Currently, agriculture uses more than 70% fresh water in the world [1]. The irrigation area and water consumption are expected to increase as the global climate becomes drier and warmer, which seriously threaten the sustainable development of global agriculture [1,2]. About 6% of cultivated land and one-fifth of irrigated land globally are salinized [3,4], which is expected to become worse by the severe salinization of more than 50% of arable land by 2050 [5,6]. In addition, climate models predict a higher incidence of extreme temperatures (i.e., extreme low temperature and extreme high temperature) in the future [7,8]. These abiotic stresses result in restricted plant cell division and growth, lower fertility, the promotion of senescence, and even cell death [9]. Both plant scientists and crop breeders are facing the great challenge of cultivating agricultural plant varieties that maintain high productivity under various environmental stresses [10]. Therefore, revealing the underlying mechanisms of plant response to abiotic stresses, which includes perceiving environmental challenges, transmitting the stress signals, and making appropriate adjustments in growth and development in order to survive and reproduce, is crucial for future agricultural development.

Perennial ryegrass (*Lolium perenne* L.), native to Europe, Asia, and Northern Africa, is a cool-season perennial grass cultivated around the world with a breeding history of more than 100 years [11,12]. Due to its outstanding forage quality, long growing season, high yield, grazing tolerance, and high palatability, perennial ryegrass is the most widely cultivated perennial gramineous forage in temperate regions [13,14,15]. Due to its prominent lawn quality, perennial ryegrass is also used as turf grass on golf courses, sports fields, and parks [16,17]. For example, it accounts for 50% of the total used land and 70% of agricultural land in the UK [18]. As a consequence of its wide distribution and perennial characteristics, perennial ryegrass is exposed to, and has to respond to, a variety of abiotic stresses. These abiotic stresses are sometimes seasonal, but increasingly unpredictable due to climate change [19]. Therefore, abiotic stresses such as drought, high salinity, and extreme temperatures are major restrictive factors in perennial ryegrass growth and management [12,17,20]. Breeding stress-resistance cultivars is considered as an important measure to mitigate the effects of abiotic stress on perennial ryegrass [17,19]. However, as perennial ryegrass is a self-incompatible species [21,22], and that plant tolerance to abiotic stresses is a complex quantitative trait involving multiple genes and complex mechanisms, breeding varieties in perennial ryegrass by conventional breeding strategies (e.g., hybrids, induced mutations, and somaclonal variation) is time-consuming and often yields unpredictable results [23,24,25]. The draft genome sequence of perennial ryegrass was reported in 2015 [26], and there is more genetic and genomic information available than other major perennial herb species [21,27]. Thus, it is widely accepted that genetic engineering is a perfect alternative for the improvement in stress resistance [25]. The knowledge of stress resistance genes and molecular mechanisms of perennial ryegrass is of great significance for cultivating new varieties with strong stress resistance.

A comprehensive review on the molecular mechanisms and signaling pathways of abiotic stress response predominantly focuses on a few annual model or crop plants [1,28,29]. However, the stress response mechanism of perennial ryegrass, which may differ to that of annual plants, has not been reported thus far. Here, we review the advances in perennial ryegrass in response to drought, salinity, and temperature stresses and focus on the understanding of the underlying survival strategies and adaptation mechanisms, especially the key stress-related genes and signal transduction systems. This review will contribute to a better understanding of the molecular mechanisms of perennial ryegrass response to abiotic stresses and provide useful information for improving the stress resistance, which is critical to agricultural and environmental sustainability.

## 2. Drought Stress

Drought is a major constraint to the growth and development of perennial ryegrass with typical symptoms including leaf senescence and desiccation, slow shoot and root growth, and even death [25,30]. Knowledge about the adaptation mechanisms of perennial ryegrass to drought stress is important for sustainable agriculture. The ability of plants to maintain growth and survival when subjected to drought stress is broadly defined as drought resistance [31]. Drought resistance is a quantitative characteristic that is determined by many genes and biochemical processes [1,32]. The identification and characterization of genes associated with drought resistance are critical in clarifying the mechanisms of perennial ryegrass adaptation to drought stress. However, the research on drought stress-related genes of perennial ryegrass is very limited, and molecular mechanisms in response to the drought environment are mostly unknown. Some drought response genes of perennial ryegrass found in limited studies are as follows. Through transcriptomic and metabolomic analyses, Foito et al. identified 38 and 15 genes with significantly increased expression under water stress in the leaves and roots, respectively. The transcripts homologous to known dehydration responsive element binding (DREB) transcription factors and aquaporins were identified [33]. The dehydration-responsive element (DRE), a 9-bp conserved sequence TACCGACAT, is essential for regulating the expression of dehydration response genes [34]. DREB transcription factors specifically combine with DRE cis-acting elements and have a vital function on conducting stress responses such as drought and salt. Transgenic ryegrass overexpressing *Arabidopsis* DREB1B shows stronger drought resistance [35]. These data suggest that DREB transcription factors may be key regulators of perennial ryegrass response to drought stress. By analyzing the transcriptome of perennial ryegrass leaves and roots under drought stress, Amiard et al. found that myoinositol inositol 1-phosphate synthase (INPS) and galactinol synthase (GOLS) regulate the content of loliose and raffinose under drought stress [36]. In addition to the transcriptional analysis, the results of the genome-wide association study (GWAS) of the drought tolerance traits in 192 perennial ryegrass cultivars distributed in 43 countries suggested that *Lolium perenne Late Embryogenesis Abundant3* (*LpLEA3*) and *Superoxide Dismutase* (*LpFeSOD*) are important for maintaining the leaf water content under drought stress [23].

Determining the role of drought-responsive genes in the adaptation of perennial ryegrass to drought stress by genetic transformation is very important in clarifying the drought resistance mechanism, as has been carried out in *Arabidopsis* and other crops [37]. It has been proven that *Lolium perenne* pyrroline-5-carboxylate synthase (LpP5CS) plays a vital role in the response to diverse stresses and is potentially a candidate gene for stress-related molecular breeding in perennial ryegrass [38]. LpP5CS includes all conserved functional sites and regions of P5CS [38,39]. The overexpression of LpP5CS in tobacco plants, especially the mutation form LpP5CSF128A without the feedback inhibition of proline, enhances the tolerance to drought stress [38]. The ubiquitin-like (Ubl) post-translational modifiers contain a subfamily designated ‘Homology to Ub1′ (HUB1), which is also known as Ubl5 [40]. Although HUB1 proteins are cognate with ubiquitins in structure, their amino acid resemblance to ubiquitins is only about 35% [40]. It is worth noting that transgenic perennial ryegrass overexpressing LpHUB1 exhibits an improved drought tolerance phenotype with a higher relative water content and growth rate under drought stress. As an ubiquitin-like modifier, LpHUB1 may regulate the protein interaction, activity, and cellular location of existing proteins in response to a stress environment [25].

MicroRNAs (miRNAs) are indispensable in the post-transcriptional regulation of target genes. Studying variations of miRNAs in ryegrass is crucial for comprehending the stress response mechanisms [41]. *miRNA408* (*miR408*), a conserved miRNA, is known to take part in multiple kinds of stress responses and has a central function in plant survival under a stress environment [42,43]. The overexpression of *miR408* in *Arabidopsis* results in an increased tolerance to salinity, low temperature, and oxidative stress, but decreased tolerance to drought and osmotic stress [44]. A recent study revealed that transgenic perennial ryegrass with heterologous expression of the rice (*Oryza sativa* L.) *miR408* gene showed improved drought tolerance, which may be due to changes in the leaf morphology and increase in the antioxidant capacity [45]. These data demonstrate that *miR408* may have different functions in *Arabidopsis* and in gramineous plants and is able to act as a potential target for genetic manipulation to improve the drought tolerance of perennial ryegrass.

**Figure 1:**
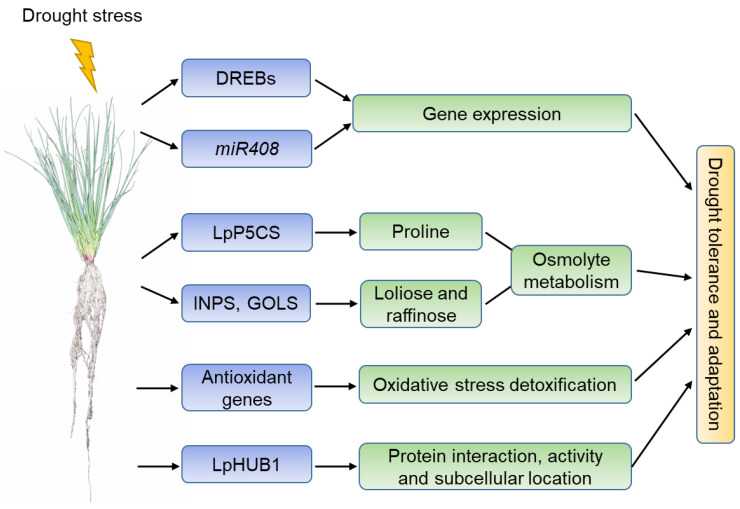
A schematic model of the perennial ryegrass response to drought stress. Abbreviations: DREB, dehydration responsive element binding; *miR408*, *miRNA408*; P5CS, pyrroline-5-carboxylate synthase; INPS, myoinositol inositol 1-phosphate synthase; GOLS, galactinol synthase; SOD, superoxide dismutase; HUB1, homology to Ub1.

Although more drought-responsive genes in perennial ryegrass should be identified, the current studies have shown that the drought stress signaling and mechanisms of perennial ryegrass are as follow: (1) Under drought conditions, DREB transcription factors regulate the expression of stress-responsive genes together with miRNAs such as *miR408*; (2) the expression of proline synthase LpP5CS and loliose- and raffinose-metabolism enzymes INPS and GOLS are differentially regulated under drought conditions, leading to changes in osmolyte metabolism and tolerance to drought stress; (3) the expression of LpSOD and other antioxidant enzymes such as POD, CAT, and APX maintains the homeostasis of reactive oxygen species (ROS) (i.e., H_2_O_2_, OH^•^, ^1^O_2_, and O^2−^) under drought conditions, and results in the adaptation of perennial ryegrass to drought stress; (4) LpHUB1, an ubiquitin-like modifier, regulates the protein interaction, activity, and cellular location of existing proteins under stress environments and promotes the adaptation of perennial ryegrass to drought stress (Figure 1).

## 3. Salt Stress

Salt stress causes harm to perennial ryegrass through ionic stress, osmotic stress, and secondary stresses including the accumulation of toxic compounds and the disruption of nutrient balance. A reduction in the shoot and root dry weight is commonly observed in cool-season gramineous grass under salinity stress [46,47]. Although a complete understanding of the gene function of perennial ryegrass is yet to be achieved, identifying the genes that play central roles in salt response and tolerance will provide new insights into the molecular mechanisms. In this section, we illustrate the expression regulation of salt-induced genes, which is helpful to comprehend the molecular mechanisms of salt tolerance in perennial ryegrass. According to previous studies, the salt-overly-sensitive (SOS) signal transduction pathway is a pivotal mechanism of salt tolerance in *Arabidopsis*. In this pathway, the calcium-binding protein, SOS3, is activated by the elevated free Ca^2+^ in cytosolic when suffering under high salinity [48]. SOS3 interacts with a serine/threonine protein kinase, SOS2, to form a SOS2/SOS3 complex, which leads to the activation of SOS1 [49,50,51]. The results of the suppression of subtractive hybridization and RT-PCR analysis in perennial ryegrass showed that approximately 22% of salt-responsive genes were related to general metabolism, 16% to interrelated protein metabolism, 12% related to signaling/transcription, and 2–4% associated with detoxification and energy transfer [52]. By RT-PCR, Liu et al. demonstrated that the expression levels of *LpSOS1* increased in the roots of perennial ryegrass but decreased in the stem under salt stress. Moreover, the expressions of *NHX1*, *TIP1*, and *PIP* were also associated with salinity tolerance in perennial ryegrass [53]. The salt tolerance of transgenic perennial ryegrass was significantly improved by the transformation of the rice vacuolar Na^+^/H^+^ antiporter OsNHX1 [54]. Furthermore, Li et al. isolated two salt-induced *P5CS* genes (*P5CS1* and *P5CS2*) from perennial ryegrass by using rapid-amplification of cDNA ends PCR (RACE-PCR). The accumulation of proline, which is significantly induced after salt treatment, is in line with the expression levels of those two *P5CS* genes under high salinity treatment [20], suggesting that both *P5CS* genes may be involved in the salt stress tolerance of perennial ryegrass.

A high salinity environment induces excessive ROS such as H_2_O_2_, OH^•^, and O^2−^, leading to organelle and tissue injury. In order to suppress the damage caused by ROS, plants usually produce more antioxidant enzymes to maintain ROS homeostasis [55]. Beyond that, Ca^2+^ and H_2_O_2_ are vital secondary messengers in plant signaling networks, triggering different physiological and molecular responses to various environmental stresses [56,57,58]. By treatments of perennial ryegrass with NaCl, exogenous Ca^2+^, and H_2_O_2_, and determination of the physiological indexes and the contents of Ca^2+^, H_2_O_2_, OH^•^, and O^2−^, Hu et al. proved that Ca^2+^ and H_2_O_2_ signaling were integrated to enhance the adaptation to stress conditions in perennial ryegrass. Ca^2+^ signaling maintains ROS homeostasis in stressed grasses by increasing the responses of antioxidant genes, proteins, and enzymes. H_2_O_2_ signaling also induces antioxidant genes but attenuates the signaling of Ca^2+^ in the root of perennial ryegrass [59]. Consistently, the results of RT-PCR suggest that the expressions of CAT, POD, APX, GPX, and GR are upregulated in perennial ryegrass under salt stress, suggesting that these antioxidant enzymes play an important role in eliminating ROS [55]. Collectively, the H_2_O_2_ and Ca^2+^ signaling of salinity response supplies a strategy for the adaptation of perennial ryegrass to salt stress. Additionally, other micromolecule metabolites are profoundly involved in salinity tolerance [60]. A study on the stress memory by the RT-PCR analysis of stress memory genes and GC–MS analysis of the metabolite profiles demonstrated that the metabolites in perennial ryegrass such as sugar and sugar alcohol regulated by trainable genes *Brown Plant Hopper Susceptibility Protein* (*PBSP*) and *Sucrose Synthase* (*SUCS*) played positive roles in the physiological changes induced by stress memory. Moreover, pre-exposure to multiple low NaCl concentrations improved the salinity response of perennial ryegrass to a subsequent worse salinity environment [61]. The application of exogenous cytokinin 6-benzylaminopurine (BAP) upregulates the gene expression of *Lolium perenne High-affinity Potassium Transporter* (*LpHKT*) and *LpMYB* and alleviates salt-induced cell damage and leaf senescence in perennial ryegrass [47]. Acetic acid is a natural endogenous substance to resist salt stress in perennial ryegrass. The results of transcriptome sequencing and acetic acid content determination under salt stress showed that the content of endogenous acetic acid gradually increased, along with the elevated expression of the key biosynthetic gene *LpPDC1*, under high salinity conditions [62].

**Figure 2:**
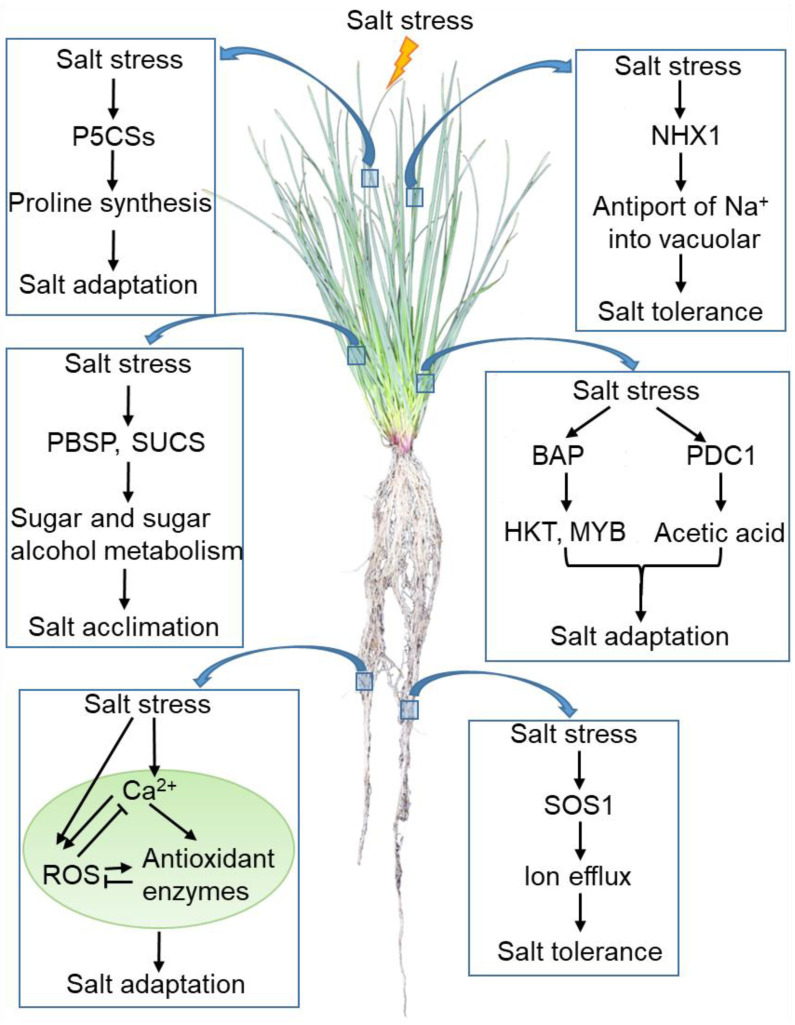
A schematic model of the perennial ryegrass response to salt stress. The arrow represents positive regulation, whereas the line ending with a bar represents negative regulation. Abbreviations: SOS1, Salt Overly Sensitive 1; NHX1, Na^+^/H^+^ antiporter 1; P5CS, Pyrroline-5-carboxylate synthase; PBSP, Brown Plant Hopper Susceptibility Protein; SUCS, Sucrose Synthase; ROS, reactive oxygen species; BAP, 6-benzylaminopurine; HKT, High-affinity Potassium Transporter; MYB, MYB transcription factor.

For the purpose of improving the tolerance of perennial ryegrass to salt stress, it is of great significance in understanding the response strategies and signal transduction processes. Although it remains unknown how Na^+^ is sensed in cellular systems and what the complete salt stress signaling network in perennial ryegrass is, the current studies show that perennial ryegrass may adopt the following strategies to adapt to high salinity stress. (1) Under salt stress, SOS1 and NHX1 are activated and mediate the efflux of ion or antiport of Na^+^ into the vacuolar, leading to the adaptation of ryegrass to salt stress. (2) Salt stress activates the expression of *P5CS* genes or *PBSP* and *SUCS*. These genes alleviate the damage of osmotic stress by regulating the synthesis of osmolytes under salt stress. *PBSP* and *SUCS* also regulate the salt acclimation by mediating the metabolism of sugar and sugar alcohol. (3) Salt stress induces the increase in cytosolic Ca^2+^ and ROS concentrations, which triggers the downstream signaling networks to enhance the adaptation of perennial ryegrass to salt stress. It is worth noting that excessive ROS is harmful. Both Ca^2+^ and H_2_O_2_ increase the expression and activity of antioxidant enzymes (e.g., CAT, POD, APX, GPX, and GR) to maintain ROS homeostasis in stressed grasses. (4) Some endogenous bioorganic micromolecules such as BAP and acetic acid also serve functions in the perennial ryegrass response to salt tolerance (Figure 2).

## 4. Cold Stress

Perennial ryegrass is a kind of perennial gramineous grass widely cultivated in temperate regions around the world. Hence, perennial ryegrass is inevitably affected by cold stress in winter and early spring every year. Improving the cold tolerance and the recovery ability of perennial ryegrass after cold stress is a key component in improving the grassland performance. The effects of cold stress on plant metabolism come from the direct inhibition of metabolic enzymes and the reprogramming of gene expression by low temperature [63]. Zhang et al. discovered by RT-PCR that *Cold-Regulated* (*COR*), *Dehydration-responsive* (*DR*), and *Ice Recrystallization Inhibition* (*IRI*) genes were upregulated after the cold treatment of perennial ryegrass [64]. Products of *COR* genes including *low-temperature induced* (*LTI*), *responsive to desiccation* (*RD*) and *early dehydration-inducible* (*ERD*), lower the cellular freezing point and allow plants to withstand extensive freeze-induced cellular desiccation [65,66,67]. IRI proteins protect membranes from physical damage by inhibiting ice crystal growth and recrystallization [64]. The overexpression of *LpIRI-a* and *LpIRI-b* in *Arabidopsis* enhances the cold tolerance with reduced ion leakage [68]. The C-repeat binding factor (*CBF*) transcriptional factors, also known as *DREB*, are critical for the plant response to cold stress [69]. Three linearly assembled CBF transcriptional factors, which are also known as DREB1B, DREB1C, and DREB1A, respectively, exist in *Arabidopsis*. These CBFs bind to cold- and dehydration-responsive DNA regulatory element (DRE) [70,71]. The constitutive expression of *CBF1*, *CBF2*, or *CBF3* in *Arabidopsis* leads to changes in the biochemical components including proline, glucose, fructose, and raffinose and enhances frost resistance [72]. Transgenic *Arabidopsis* with the overexpression of *CBF1*/*DREB1B* or *CBF3*/*DREB1A* possesses the constitutive expression of downstream cold-inducible genes and improved low temperature resistance. *CBF2*/*DREB1C* ensures the transient expression of *CBF1*/*DREB1B* and *CBF3*/*DREB1A* by negatively regulating them to guarantee the appropriate induction of downstream genes [73]. By Southern and Northern analyses, Zhao et al. proved that similar to *CBF* genes in the great majority of plants, *LpCBF3*, a *CBF* homologous gene derived from a cold-resistant perennial ryegrass, contains the sequence for nuclear localization signal (NLS), the APETALA2 (AP2) DNA-binding domain, and the acidic activation motif [74]. After the identification of *LpCBF3*, five new *CBF* genes (*LpCBFI*, *II*, *III*, *IV*, and *V)* were identified in perennial ryegrass by using RACE-PCR [75]. *LpCBF3* is rapidly upregulated in response to exposure to 4 °C [74]. Overexpression of *LpCBF3* in *Arabidopsis* evokes the expression of *COR15a* and *RD29A*, which have been proven to be the target *COR* genes of *DREB1A*/*CBF3* in *Arabidopsis*. The *Arabidopsis* transformed with *LpCBF3* displayed a phenotype of stunting, later flowering, and increased freezing tolerance [74,76]. In addition, the transformation of the *Arabidopsis DREB1A*/*CBF3* gene in perennial ryegrass improved the freezing stress resistance due to the enhanced activities of antioxidant enzymes such as superoxide dismutase (SOD) and peroxidase (POD) [77]. These studies suggest that *CBF* genes may serve as useful candidates for the improvement in cold tolerance in perennial ryegrass.

On the other hand, protein metabolism undergoes dramatic changes to resist low temperature including selective up- or downregulation of protein synthesis. For instance, antifreeze proteins (AFPs) are induced and accumulated in the apoplast space of plant tissues under a cold environment. AFPs are the sort of proteins present in a series of over-wintering plants that play functions in a freezing-tolerant strategy [78]. AFP proteins possess the ability to adsorb to the surface of the ice crystals, protecting plant tissues from mechanical stress induced by the formation of ice crystals [79]. *Lolium perenne* has no ability to endure cold temperatures below the crystallization point. By analyzing the LpAFP expression profile and protein characterization, Lauersen et al. found that *LpAFP* plays a role in keeping perennial ryegrass away from the damage caused by a frosty environment [80]. Furthermore, a previous study on the transformation of *LpAFP* in tomato showed that the *LpAFP* gene endues cold resistance in transgenic tomato plants. Frosty stress gives rise to physiological damage in wild type tomato plants, while the transgenic plants maintain normality [81].

**Figure 3:**
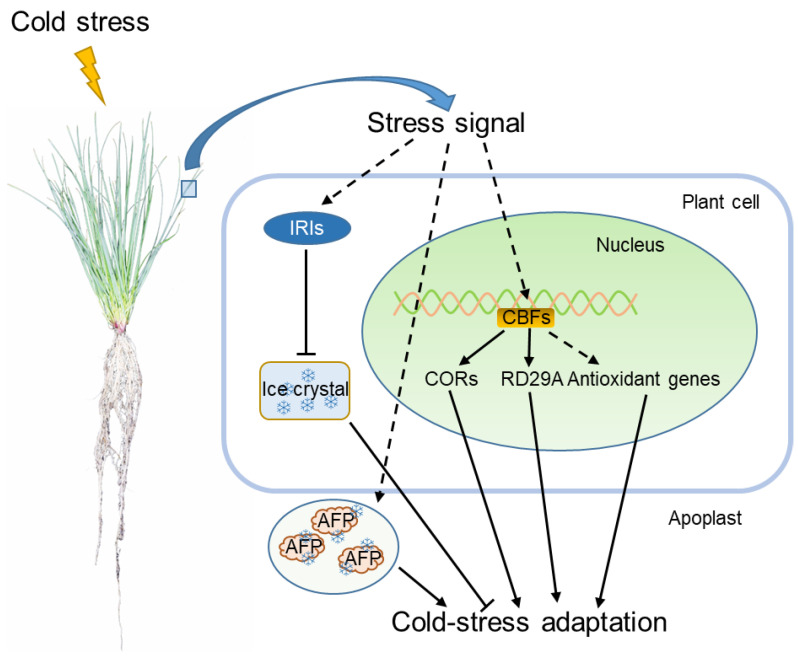
A schematic model of the perennial ryegrass response to cold stress. The arrow represents positive regulation, whereas the line ending with a bar represents negative regulation. Snowflake graphic represents ice crystals. Abbreviations: CBF, C-repeat binding factor; COR, Cold-Regulated; IRI, Ice Recrystallization Inhibition; AFP, antifreeze proteins.

Based on the progress achieved in the research on perennial ryegrass response to cold stress, we can conclude that the strategies and signaling pathways of perennial ryegrass to adapt to low temperature stress are as follows. (1) LpCBF transcription factors activated by low temperature stress regulate the expression of downstream genes such as *CORs* and *RD29A* and facilitate the adaptation of ryegrass to cold stress. Furthermore, LpCBFs regulate ROS (mainly is H_2_O_2_ and OH^•^) homeostasis under cold stress by affecting the antioxidant enzymes. (2) LpIRI-a and LpIRI-b inhibit ice crystal growth and recrystallization in plant cells to alleviate the mechanical damage of membranes and improve the cold tolerance of perennial ryegrass. (3) The apoplast localized LpAFP adsorbs to the surface of the ice crystals to protect the tissues of perennial ryegrass from mechanical damage caused by ice formation (Figure 3).

## 5. Heat Stress

Perennial ryegrass is a heat-stress susceptible grass species. Its growth and development often suffer from extreme high temperature, which becomes more frequent with climate warming [12]. Identifying the genes regulating heat tolerance and clarifying the adaptive mechanism of perennial ryegrass will provide insights for the improvement in the heat tolerance of perennial ryegrass. The heat stress response is regulated by an integrated pathway comprising many transcription factors and signaling molecules in plants [41]. The homeodomain leucine zipper (HD-Zip) transcription factor family plays an important role in regulating plant development and in coping with abiotic stresses including heat stress. A total of 13 HD-Zip I genes were identified in the transcriptome of perennial ryegrass by using the tblastn program with HD-Zip proteins in rice as query sequences. The RT-PCR results suggest that the expression levels of *LpHOX6*, *LpHOX8*, and *LpHOX24* (HD-Zip I transcription factor in *Lolium perenne*) are negatively correlated while *LpHOX21* is positively correlated with the heat tolerance in perennial ryegrass [82]. Cytochrome P450 (P450) catalyzes a variety of monooxygenation and hydroxylation reactions in plant cell and take part in the plant response to abiotic stress [83,84]. By using sodium bisulfite sequencing and methylation analysis, Dai et al. revealed that the perennial ryegrass P450 gene *LpCYP72A161* is epigenetically regulated under heat stress. The methylation of CpG islands in *LpCYP72A161* exon 1 is significantly reduced when facing heat stress, resulting in a higher transcription. This research provides new insights into the epigenetic regulation of perennial ryegrass genes under temperature stress [85].

To resist heat stress, heat shock proteins (HSPs) are induced and act as molecular chaperones to prevent protein denaturation, which is a process controlled by the heat stress transcription factors (heat shock factor, HSF) [86]. Wang et al. analyzed the gene expression changes of perennial ryegrass under temperature stress by RNA-Seq, and found that the subfamilies of *HSFA* and *HSFB* were related to the acclimation of perennial ryegrass to heat tolerance [87]. Among the genes of the subfamily *HSFC*, the *LpHSFC1b* gene is significantly induced after heat treatment and acts as a core regulator. Heterologous expression of *LpHSFC1b* in *Arabidopsis* enhances the plants’ heat tolerance. The contents of electrolyte leakage (EL) and malondialdehyde (MDA) in *Arabidopsis* transformed with *LpHSFC1b* decreased, and the expression of the heat stress-responsive genes increased significantly [11]. Furthermore, *HSFA1b* is also a positive regulator of heat stress and the overexpression of *HSFA1b* increased heat tolerance in *Arabidopsis* [88,89,90]. These data suggest that *HSFs* may play a positive regulatory roles in the heat stress response of perennial ryegrass.

When exposed to long-term heat stress, the leaf chlorophyll (Chl) content of perennial ryegrass greatly decreased, which led to the leaf senescence. Reduced Chl content is mainly due to the acceleration of Chl degradation rather than the inhibition of Chl biosynthesis under heat stress [91]. The determination of molecular markers related to the genetic variation of heat tolerance in different perennial ryegrass germplasm revealed that the expression levels of four Chl catabolism genes, *LpNYC1*, *LpNOL*, *LpSGR*, and *LpPPH* were associated with the tolerance to heat stress, and elevation of their expression levels enhanced the heat resistance of ryegrass [12]. Recently, Zhang et al. blocked the enzymatic catabolism of chlorophyll a (Chl a) by inhibiting the expression of *LpSGR* using RNAi, an essential Chl catabolic enzyme, leading to exacerbation of the degree of the heat-induced photosynthetic system II (PSII inhibition), ROS (such as H_2_O_2_ and OH^•^) homeostasis, and chloroplast breakdown [92]. These studies have demonstrated that the Chl catabolic proteins (i.e., LpNYC1, LpNOL, LpSGR, and LpPPH modulated Chl catabolism) are one of the mechanisms by which perennial ryegrass adapts to heat stress.

In addition to the up- or downregulation of heat tolerance genes, hormones such as abscisic acid (ABA), cytokinin (CK), and melatonin (MT) also undertake major functions in the heat tolerance of perennial ryegrass. The application of exogenous MT to perennial ryegrass could alleviate the growth inhibition and leaf senescence caused by heat stress, which is manifested as a significant increase in the tiller number, plant height, leaf Chl content, and the reduction in the transcript levels of the senescence-related genes *LpSAG12.1* and *Lph36* [93]. *LpABI3* and *LpABI5*, which encode key transcription factors in the ABA signaling pathway, are also transcriptionally upregulated by heat stress but repressed by MT treatment, suggesting a crosstalk between these two signaling pathways to prevent the excessive inhibition of ABA on plant growth under heat stress. As transcription factors in the CK signaling pathway, the transcription of the B-type ARR genes *LpARR1* and *LpARR10* is repressed by heat stress, while MT treatment alleviates this inhibitory effect [93]. Taken together, MT, ABA, and CK cooperatively regulate the adaptability of perennial ryegrass to high temperature stress.

**Figure 4:**
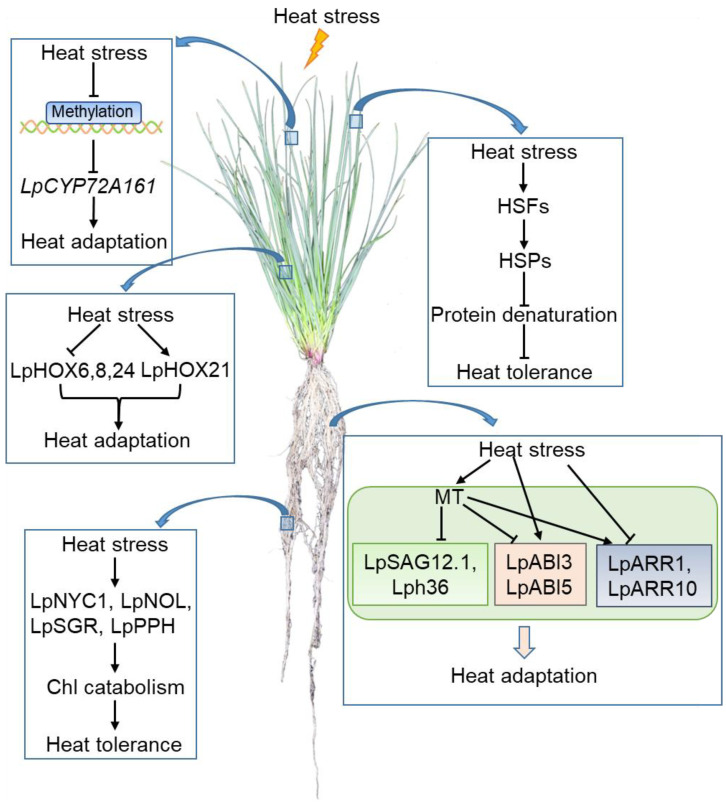
A schematic model of the perennial ryegrass response to heat stress. The arrow represents positive regulation, whereas the line ending with a bar represents negative regulation. Abbreviations: Chl, chlorophyll; HSF, heat shock factor; HSP, heat shock protein; MT, melatonin.

Therefore, some progress has been made by researchers on the heat stress response in perennial ryegrass. Based on this, we can conclude that the strategies and signaling pathways of perennial ryegrass to adapt to heat stress are as follows. (1) Under extremely high temperatures, the methylation of CpG islands in *LpCYP72A161* is reduced, resulting in the change in its expression level and the tolerance of perennial ryegrass to heat stress. (2) The variations in the expression of negative related HD-Zip I transcription factors LpHOX6, LpHOX8, LpHOX24m and positive related transcription factor LpHOX21 affect the adaptability of perennial ryegrass to heat stress. (3) Under extremely high temperatures, LpNYC1, LpNOL, LpSGR, and LpPPH enhance the heat resistance of ryegrass by affecting the catabolism of Chl. (4) Heat stress induces the expression of HSFs such as LpHSFC1b. These HSFs activate the expression of HSPs to prevent protein denaturation and enhance the heat resistance of ryegrass. (5) The phytohormone MT positively regulates the adaptability of perennial ryegrass to high temperature stress by inhibiting the expression of *LpSAG12.1* and *Lph36*, affecting the signaling pathways of ABA and CK under high temperature (Figure 4).

## 6. Conclusions and Future Perspectives

With global climate change, environmental problems such as drought, salinity, and extreme temperatures are becoming increasingly more severe. With the development of life science, great progress has been made in the stress resistance research of perennial ryegrass, and some key stress-related genes have been identified. This work revealed some of the molecular mechanisms and signal pathways of the perennial ryegrass response to abiotic stress. However, our understanding of the abiotic stress adaptation mechanisms of perennial ryegrass is still seriously deficient and fragmented, and a complete stress signaling network including stress perception, signal transduction, and the adjustment of gene expression and metabolism is yet to be achieved.

In order to comprehensively clarify the abiotic stress molecular mechanisms of perennial ryegrass and improve its stress resistance, future research should pay attention to the following items. (1) The identification and functional study of key components of abiotic stress signal transduction in perennial ryegrass such as stress sensors, kinases, and transcription factors remain important but challenging goals for further research. (2) Technologies such as genome editing, high-throughput phenotypic analysis, and the genetic transformation of perennial ryegrass need to be further developed. At present, genetic transformation of perennial ryegrass only works in a few varieties. The gene editing technology of perennial ryegrass is also in its infancy. The development of these technologies will contribute to the dissection of molecular mechanisms and the improvement in perennial ryegrass. (3) Because the stress resistance improvement of perennial ryegrass should not restrict its growth and development, it is important to understand the crosstalk between the stress signaling pathway and the growth and development signaling pathway. (4) Most of the knowledge about the response of perennial ryegrass to abiotic stress has been obtained in the laboratory. How to use them to cultivate high resistant varieties grown in the field is also a challenge.

## Data Availability

Not applicable.
